# Dr. William S. Searle

**Published:** 1911-01

**Authors:** 


Dr. William S. Searle, of Brooklyn, a medical graduate of the University of Pennsylvania, 1859, died at his home October 30, 1910, aged 77 years. He was a member for three years of the State Board of Medical Examiners, of which he was vice-president and examiner in diagnosis.

